# The Fatty Liver, Cirrhosis, and Liver Cancer Study (TENDENCY): Protocol for a Multicenter Case-Control Study

**DOI:** 10.2196/44264

**Published:** 2023-05-31

**Authors:** Yaqza Hussain, Ayman Bannaga, Neil Fisher, Ashwin Krishnamoorthy, Peter Kimani, Ahmad Malik, Maria Truslove, Shivam Joshi, Megan Hitchins, Abdullah Abbasi, Christopher Corbett, Matthew Brookes, Harpal Randeva, Nwe Ni Than, Ramesh P Arasaradnam

**Affiliations:** 1 University Hospitals Coventry & Warwickshire Coventry United Kingdom; 2 Russells Hall Hospital Dudley United Kingdom; 3 Warwick Medical School University of Warwick Warwick United Kingdom; 4 Pakistan Kidney and Liver institute and Research Center Lahore Pakistan; 5 Cedars-Sinai Medical Center Los Angeles, CA United States; 6 New Cross Hospital Wolverhampton United Kingdom; 7 Leicester Cancer Research Centre University of Leicester Leicester United Kingdom

**Keywords:** hepatocellular cancer, cirrhosis, methylated septin 9, urinary volatile organic compounds, urinary peptides, fatty liver, fatty liver disease, hepatitis, liver cancer

## Abstract

**Background:**

Hepatocellular cancer (HCC) is associated with high mortality, and early diagnosis leads to better survival. Patients with cirrhosis, especially due to nonalcoholic fatty liver disease and viral hepatitis, are at higher risk of developing HCC and form the main screening group. The current screening methods for HCC (6-monthly screening with serum alpha fetoprotein and ultrasound liver) have low sensitivity; hence, there is a need for better screening markers for HCC.

**Objective:**

Our study, TENDENCY, aims to validate the novel screening markers (methylated *septin 9*, urinary volatile organic compounds, and urinary peptides) for HCC diagnosis and study these noninvasive biomarkers in liver disease.

**Methods:**

This is a multicenter, nested case-control study, which involves comparing the plasma levels of methylated *septin 9* between confirmed HCC cases and patients with cirrhosis (control group). It also includes the comparison of urine samples for the detection of HCC-specific volatile organic compounds and peptides. Based on the findings of a pilot study carried out at University Hospital Coventry & Warwickshire, we estimated our sample size to be 308 (n=88, 29% patients with HCC; n=220, 71% patients with cirrhosis). Urine and plasma samples will be collected from all participants and will be frozen at –80 °C until the end of recruitment. Gas chromatography–mass spectrometry will be used for urinary volatile organic compounds detection, and capillary electrophoresis–mass spectrometry will be used for urinary peptide identification. Real-time polymerase chain reaction will be used for the qualitative detection of plasma methylated *septin 9*. The study will be monitored by the Research and Development department at University Hospital Coventry & Warwickshire.

**Results:**

The recruitment stage was completed in March 2023. The TENDENCY study is currently in the analysis stage, which is expected to finish by November 2023.

**Conclusions:**

There is lack of effective screening tests for hepatocellular cancer despite higher mortality rates. The application of more sensitive plasma and urinary biomarkers for hepatocellular cancer screening in clinical practice will allow us to detect the disease at earlier stages and hence, overall, improve HCC outcomes.

**International Registered Report Identifier (IRRID):**

DERR1-10.2196/44264

## Introduction

### Background

Hepatocellular cancer (HCC) has been the sixth most common cancer worldwide and the third most frequent cause of cancer-related deaths around the world in 2020 [1]. There has been a significant increase in the incidence of HCC in the United Kingdom and worldwide. Cirrhosis is the most potent risk factor for HCC and hence constitutes the major screening group for HCC globally. Cirrhosis can be caused by chronic viral hepatitis (B and C), chronic alcohol abuse, inherited metabolic diseases such as genetic haemochromatosis, or in some cases alpha-1-antitrypsin deficiency. The acquired metabolic disorder, nonalcoholic fatty liver disease (NAFLD), has also emerged as an important risk factor. NAFLD is a manifestation of the metabolic syndrome and is associated with type 2 diabetes mellitus and obesity. NAFLD affects around 20% to 30% of the adult population. Growing evidence suggests an increased HCC incidence in patients with NAFLD, and it will become the leading cause of HCC in the developed world [2,3].

The current screening strategy for HCC in the United Kingdom in established cirrhotic and high-risk groups is 6-month (twice a year) ultrasound surveillance scans and serum alpha fetoprotein (AFP) measurement. However, there are significant limitations associated with this approach. The sensitivity of AFP for HCC detection is only 60%, and the specificity is 87.7%. Previously, any attempts to increase the cutoff to increase the specificity had resulted in a lower sensitivity [4]. In fact, previous large-scale trials had shown that up to half of patients with primary liver cancer are AFP negative [5]. Hence, it is far from ideal as a screening test for HCC surveillance. Ultrasound liver is very much operator dependent, and again, the sensitivity remains modest around 63% for an early-stage HCC case [6].

There is always a significant risk of missing early-stage HCC or late diagnosis of HCC due to the poor sensitivity of the current screening modalities. Unfortunately, the late diagnosis of HCC is associated with overall poor outcomes, with a median survival of around 6 to 20 months post diagnosis [7]. Early diagnosis can be associated with curative options, including surgical resection, liver transplantation, and radiofrequency ablation. However, the chances of the earlier diagnosis rely mostly on better screening.

Current methods used to diagnose cirrhosis and NAFL have limitations. Transient elastography (TE) and enhanced liver fibrosis tests are used to check the presence of fibrosis, with TE also giving estimations of fatty changes in liver. However, they both have limitations, with enhanced liver fibrosis being a proprietary test [8] at a high cost (and with data coming from observational studies), and TE being operator dependent and nonconclusive in patients with a high BMI [9]. Hence, better noninvasive diagnostic tests are required for NAFLD and cirrhosis diagnosis and HCC screening.

### Potential New Screening Markers for HCC and Liver Disease for Further Evaluation in TENDENCY

#### Methylated Septin 9

Studies on colorectal cancer have identified that methylation of the *septin 9* gene occurs during carcinogenesis and is released into the plasma, which leads to the development of blood-based *septin 9* gene methylation assays [10]. A study of 304 HCC tissue samples showed that methylated *septin 9* *(mSEPT9)* is a significant epi-driver gene in liver carcinogenesis [11]. He et al [12] conducted a case control study (64 patients with HCC, 44 patients with liver cirrhosis, and 298 healthy individuals) and demonstrated an overall plasma *mSEPT9* sensitivity of 76.7% for the detection of HCC. However, further work is required to validate these findings in screening a high-risk cohort [12].

Li et al [13] showed plasma *mSEPT9* to have a sensitivity of 82% and a specificity of 96% for HCC detection in a similar study carried out in Beijing (including 104 patients with HCC, 95 patients at risk of the disease, and 174 healthy controls).

A proof-of-concept study done as part of the TENDENCY study at University Hospital Coventry & Warwickshire (UHCW) with 141 participants (including 39 HCC cases) showed *mSEPT9* to have a sensitivity of 89% and a specificity of 81% (in comparison to an AFP sensitivity of 50% and a specificity of 97%) [14]. Thus, *mSEPT*9 has a far better sensitivity than AFP. However, in order to prove its applicability over the screening population, we require validation in a larger cohort of HCC cases against a control group of patients with cirrhosis (which is the major screening group in clinical practice).

#### Volatile Organic Compounds

Volatile organic compounds (VOCs) are endogenous and exogenous compounds with a lower boiling point and high vapor pressure. Endogenous VOCs are predominantly produced as a result of fermentation of nonstarchy polysaccharides and fibers in gut. These compounds then permeate the bowel wall and enter the portal circulation. Liver then plays further role in the metabolism of these compounds via various enzymes. The end products are subsequently excreted in various body secretions [15]. In addition, several volatile organic compounds are produced in liver as a result of complex metabolic processes (including lipid peroxidation, dextrose metabolism, and pathways involving cytochrome P450 enzymes). Any disruption in these metabolic pathways and possible oxidative stress can lead to fluctuations in the levels of relevant VOCs [16]. This fact has formed the basis for further studies involving the identification of various VOCs as possible biomarkers for liver diseases.

The UHCW research group conducted a pilot study that provides proof of concept for further research into the role of VOCs in NAFLD. The aim of the study was to determine whether urinary VOCs could distinguish between various stages of fatty liver disease (NAFLD, nonalcoholic steatohepatitis [NASH], and NASH-C) and controls. The results suggested that urinary VOCs can help distinguish between NASH and NAFLD [17].

The analysis of urinary VOCs has a potential role in the screening of HCC. VOCs can be both released and metabolized by HCC cells. A pilot study at UHCW (as part of the TENDENCY project) has used gas chromatography–ion mobility spectrometry and gas chromatography–time-of-flight mass spectrometry to identify significant VOCs associated with HCC and liver fibrosis. The gas chromatography–Ion mass spectrometry data separated patients with HCC from the patients with fibrosis with an area under the receiver operating characteristic curve of 0.97. Seven compounds were identified to be provisionally associated with HCC from the gas chromatography–time-of-flight mass spectrometry data set. The compounds included 4-methyl-2,4-bis (p-hydroxyphenyl)pent-1-ene (2TMS derivative), 2-butanone, 2-hexanone, benzene, 1-ethyl-2-methyl-, 3-butene-1,2-diol, 1-(2-furanyl)-, bicyclo (4.1.0) heptane, 3,7,7-trimethyl-, [1S-(1a,3β,6a)]-, and sulpiride. However, the sample size was small, with 20 HCC and 38 non-HCC cases, and as a result, quantification was not possible [18] (Table 1).

**Table 1:** Volatile organic compound (VOCs) as potential hepatocellular cancer (HCC) biomarkers [18].

VOC identified during GC-TOF-MS^a^	Change in HCC
4-Methyl-2,4-bis(p-hydroxyphenyl)pent-1-ene,2TMS derivative	Lower
2-Butanone	Higher
2-Hexanone	Lower
Benzene, 1-ethyl-2-methyl-	Lower
3-Butene-1,2-diol, 1-(2-furanyl)-	Lower
Bicyclo[4.1.0]heptane, 3,7,7-trimethyl-, [1S-(1a,3ß,6a)]-	Lower
Sulpiride	Lower

^a^GC-TOF-MS: gas chromatography–time-of-flight mass spectrometry.

#### Urinary Peptides

Significant alterations in protein expression occur during carcinogenesis. These altered proteins then play an important role in tumor cells proliferation via the modification of signaling and other cellular metabolic pathways. Further characterization of protein alterations has shown a specific association for certain malignancies [19]. Hence, studies are being conducted to evaluate the role of these altered proteins as possible cancer biomarkers.

Several proteomics studies involving capillary electrophoresis coupled to mass-spectrometry (CE-MS) have demonstrated a good diagnostic potential of urinary peptide biomarkers [20]. A pilot study (joint collaboration of the United Kingdom and Germany) has shown that peptides specific to HCC can be identified by urinary CE-MS analysis. This study successfully identified a set of 31 urinary peptides showing an overall sensitivity of 79.5% for HCC. Further validation studies are required on a larger cohort of HCC urine samples [21].

### Aims and Objectives

The aim of this study is to investigate the metabolic function in patients with liver disease and to identify the biomarkers in liver disease.

#### Primary Objectives

Our primary aim was to study the metabolome of patients with cirrhosis, NAFLD, and HCC to identify novel noninvasive diagnostic biomarkers.

#### Secondary Objectives

We also aimed to achieve the following:

1. Characterize the urinary VOC biological signatures to identify specific chemicals that can be used as biomarkers in HCC.
2. Identify a specific urinary protein biomarker in HCC via CE-MS.
3. Study blood for nuclear markers and compounds that can be used as diagnostic tests in HCC.

## Methods

### Overview

This is a multicenter case-control study comparing patients with HCC to patients with cirrhosis. Participants in the United Kingdom will be recruited from UHCW, Russells Hall Hospital, New Cross Hospital, and Royal Stoke University Hospital. These hospitals cover the majority of West Midlands and North Midlands in the United Kingdom, providing comprehensive liver services. In addition, participants will also be recruited from Pakistan Kidney and Liver Institute and Research Center.

The participants in this study will require one visit only. Following posting the participant information sheet and a phone call, the participant will be given an option of either attending a specified research clinic or meeting after a routine clinic appointment arranged for their care in UHCW. Urine and blood samples for the study will be collected during that appointment.

For recruitment, we will be identifying positive HCC cases through HCC multidisciplinary team meetings as per the international guidelines (inclusion criteria will include radiological or histological confirmation) [2,22]. We will recruit our control group of patients with cirrhosis (inclusion criteria comprises radiological or clinical confirmation) via our cirrhotic follow-up clinics.

Patients under the age of 18 years or pregnant or those unable to give consent will be excluded from the study.

Written informed consent will be taken by a member of the research team. Before taking consent, the patient will be given adequate time to read the participant information sheet and will be given the opportunity to ask any questions they may have. Baseline data (as mentioned in Table 2) will be collected upon the recruitment of the participants prior to the collection of samples. Participant flowsheet throughout the study can be seen in Textbox 1.

**Table 2:** Schedule of events.

Procedure	Screening	Appointment	Post appointment
Eligibility check	X		
Relevant participation information sheet and invitation letter posted to participant	X		
Participant contacted to confirm their interest in taking part	X		
Patient attends routine appointment or attends dedicated research clinic		X	
Informed consent		X	
Demographic data (eg, date of birth and sex)		X	
Medical history		X	
Current medications or treatment modalities planned or done		X	
Height and weight		X	
Taking blood sample		X	
Taking urine sample		X	X
Collecting relevant clinical data done as part of the standard clinical care		X

**Participant flowsheet throughout the study.**

- Patient screened
- Confirmed patient diagnosis and eligibility
- Postinvitation letter and relevant participant information sheet
- Phone call to confirm if interested in participating
- Patient attends routine appointment or attends dedicated research clinic
- Patient approached by member of research team on the day of recruitment
- Informed consent taken
- Blood and urine sample taken
- If patient was unable to provide urine sample on the day, an option to send it via post will be provided.

### Sample Size Calculation

Definitive sample size calculations are difficult as the technologies used are novel with little available literature for comparison. However, we have estimated our sample size based on the preliminary findings of the TENDENCY pilot study conducted from 2019 to 2021.The pilot study conducted at UHCW (as part of TENDENCY) showed the sensitivity and specificity of *mSEPT9* as 89% and 81%, respectively, for detecting HCC in patients with cirrhosis [14]. Based on that, we have estimated our required total sample size using the method used by Buderer [23] to be N=308, with 88 (29%) patients with HCC and 220 (71%) patients with cirrhosis (considering a 10% dropout rate for HCC). The prevalence has been ascertained using cumulative 5-year incidence of HCC as 30% in certain groups of cirrhotic patients [24].

ME = half the 95% CI =0.05

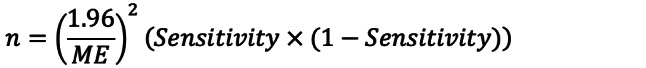

Total sample size = n/prevalence

### Storage and Analysis of Samples

After collection, urine specimens will be frozen at –80 °C in the tissue bank for later analysis. Prior to analysis, they will be left to thaw in a laboratory fridge overnight and aliquoted into appropriate sample bottles.

#### Urinary Volatile Organic Compounds

The detection of the urinary VOCs will consist of sample concentration with a sample containment system, transfer of the VOCs from the air sample to the analytical device with an odor-sampling system, and detection and identification of each VOC with proprietary instrument for analysis. Gas chromatography–Ion mass spectrometry will identify different VOCs. The data generated will then be digitized and processed by computer software and subjected to principal-components analysis to ascertain whether there are differences between the different urine samples.

#### Urinary Proteomic Analysis

For the proteomic analysis, urine samples will be processed using the CE-MS to help identify proteins specifically linked to HCC. The principal stages involved in the process would include the removal of large molecular proteins, CE-MS analysis, CE-MS data processing, and support vector machine processing and classification.

#### Plasma Analysis for Methylated Septin 9

Blood samples will be centrifuged at 5000 revolutions per minute for 15 minutes, and at least 1 milliliter plasma will then be decanted in a separate aliquot. The plasma samples will then be frozen at –80 °C until the end of recruitment.

Plasma samples will then be analyzed using real-time polymerase chain reaction with a fluorescent hydrolysis probe for the methylation-specific detection of the *septin 9* DNA target (*mSEPT9*); this automated real-time polymerase chain reaction test is aimed at a qualitative detection.

### Study Overview

UHCW is the sponsor for the study and provides indemnity for the study. An authorized representative of the sponsor has approved the final version of this protocol with respect to the study design, conduct, data analysis, interpretation, as well as plans for the publication and dissemination of the results.

### Monitoring, Audit, and Inspection

The study will be monitored by the Research & Development department at UHCW as representatives of the sponsor to ensure that the study is being conducted as per protocol, adhering to Research Governance and Good Clinical Practice. The approach to and extent of monitoring will be specified in a study monitoring plan determined by the risk assessment undertaken prior to the start of the study.

### Ethics Approval

TENDENCY has been approved by Northeast-York Ethics Committee (aproval number: 19/NE/0213). The Integrated Research Application System (IRAS) number for the study is 260179, and the National Institute for Health and Care Research (NIHR) portfolio ID is 42438. All the investigators will be granted authorship in the final study reports.

### Data Protection and Patient Confidentiality

This study will comply with the current data protection regulations, and regular checks and monitoring will be undertaken by the UHCW Research & Development department, National Health Services to ensure compliance. Participants will be assigned a unique identifier upon enrollment into the study to allow link anonymization of patient-identifiable data. Access to patient-identifiable data will be restricted to members of the study coordination team, and electronic data will be stored on password-protected, encrypted drives.

## Results

The recruitment stage was completed in March 2023. The TENDENCY study is currently in the analysis stage, which is expected to finish by November 2023. The results from TENDENCY will be published in medical journals and will be of specific interest to clinicians managing chronic liver disease. The results may form the basis for a possible change in the screening pathways for cirrhosis, NAFL disease, and hepatocellular cancer.

## Discussion

The lack of more sensitive screening tests for HCC has prompted a search for the novel biomarkers. The noninvasive blood tests and urine tests can serve as an easier and more effective method of the close surveillance of patients at a high risk for liver disease. The findings of the TENDENCY study will provide a basis of formulating better pathways for HCC screening. This is the first large-scale UK multicenter study that incorporates both urinary and plasma biomarker testing for HCC screening. The findings from this study can provide a basis for the development of assays incorporating a combination of biomarkers for enhanced early-stage HCC detection.

Based on the findings of the study, the next step will be to study the plasma *mSEPT9* in posttreatment cohorts to study its prognostic value.

The application of more sensitive plasma and urinary biomarkers for hepatocellular screening in clinical practice could allow us to detect HCC at earlier stages and overall improve the outcomes.

## Abbreviations

AFP: alpha fetoprotein
CE-MS: capillary electrophoresis coupled to mass spectrometry
HCC: hepatocellular cancer
*mSEPT9*: methylated septin 9
NAFLD: nonalcoholic fatty liver disease
NASH: nonalcoholic steatohepatitis
TE: transient elastography
UHCW: University Hospital Coventry & Warwickshire
VOC: volatile organic compound

## Data Availability

The data sets generated during this study are not publicly available as the study is still ongoing and sample analysis has not taken place.
